# Evaluation of Novel ^64^Cu-Labeled Theranostic Gadolinium-Based Nanoprobes in HepG2 Tumor-Bearing Nude Mice

**DOI:** 10.1186/s11671-017-2292-5

**Published:** 2017-09-06

**Authors:** Pengcheng Hu, Dengfeng Cheng, Tao Huang, Anna B. Banizs, Jie Xiao, Guobing Liu, Quan Chen, Yuenan Wang, Jiang He, Hongcheng Shi

**Affiliations:** 1 Department of Nuclear Medicine, Zhongshan Hospital, Fudan University, Shanghai, 200032 China; 2 Shanghai Institute of Medical imaging, Shanghai, 200032 China; 3 Shanghai Institute of Nuclear Medicine, Shanghai, 200032 China; 4 Department of Radiology and Medical Imaging, University of Virginia, 480 Ray C Hunt Dr., PO Box 801339, Charlottesville, VA 22903 USA; 5 Department of Radiation Oncology, University of Virginia, Charlottesville, VA 22903 USA

**Keywords:** Radiotherapy, Nanoparticles, Cancer, Molecular imaging, PET, MRI

## Abstract

Radiation therapy of liver cancer is limited by low tolerance of the liver to radiation. Radiosensitizers can effectively reduce the required radiation dose. AGuIX nanoparticles are small, multifunctional gadolinium-based nanoparticles that can carry radioisotopes or fluorescent markers for single-photon emission computed tomography (SPECT), positron emission tomography (PET), fluorescence imaging, and even multimodality imaging. In addition, due to the high atomic number of gadolinium, it can also serve as a tumor radiation sensitizer. It is critical to define the biodistribution and pharmacokinetics of these gadolinium-based nanoparticles to quantitate the magnitude and duration of their retention within the tumor microenvironment during radiotherapy. Therefore, in this study, we successfully labeled AGuIX with ^64^Cu through the convenient built-in chelator. The biodistribution studies indicated that the radiotracer ^64^Cu-AGuIX accumulates to high levels in the HepG2 xenograft of nude mice, suggesting that it would be a potential theranostic nanoprobe for image-guided radiotherapy in HCC. We also used a transmission electron microscope to confirm AGuIX uptake in the HepG2 cells. In radiation therapy studies, a decrease in ^18^F-FDG uptake was observed in the xenografts of the nude mice irradiated with AGuIX, which was injected 1 h before. These results provide proof-of-concept that AGuIX can be used as a theranostic radiosensitizer for PET imaging to guide radiotherapy for liver cancer.

## Background

Hepatocellular carcinoma (HCC) is one of the most common malignant tumors in the world. There were 782,500 newly diagnosed liver cancer cases and 745,500 liver cancer deaths in 2012, of which 70 to 90% were HCC [1]. Most HCC patients are classified as an advanced stage or terminal stage when they are first diagnosed, so only 20–25% of the patients are suitable for curative treatment [2, 3]. Therefore, the treatment of liver cancer requires a comprehensive multidisciplinary treatment that includes radiotherapy as a major clinically viable technique [4].

One of the major limitations of radiotherapy for HCC patients is radiation-related toxicity to the surrounding normal liver tissue. With the increasing dosage, the incidence of radiotherapy complications, including radiation-induced liver disease (RILD), is a serious threat to patients’ lives [5]. One of the strategies to avoid this issue is to use radiosensitizers that can accumulate in the tumor tissue to increase tumor cell sensitivity to radiation so that tumor cells are more likely to be killed by lower doses of radiation [6].

In 2013, Mignot et al. constructed a novel type of multifunctional gadolinium nanoparticle, AGuIX, which is small in diameter (about 5 nm), reported to be quickly excreted by the kidneys [7], and can be conjugated to radioactive or fluorescent labels for SPECT, PET, MRI, or fluorescence imaging. Because these nanoparticles carry a high number of gadolinium (atomic number 64), they can be used as tumor radiotherapy sensitizers [8]. A number of studies have shown that AGuIX nanoparticles increased the sensitivity of tumor cells to radiation therapy in various tumor cells (including radiation-resistant cell lines) in vitro. Sensitizing enhancement ratios (SER) were observed in the range 1.1 to 2.5 [8]. Given the much lower hepatic background of AGuIX compared with the high tumor uptake of AGuIX in most tumor models due to the enhanced permeability and retention (EPR) effect, this type of nanoparticle has a great potential to be developed into an ideal radiotherapy sensitizer for HCC [9].

These AGuIX nanoparticles have mainly been developed for MRI-guided radiotherapy (RT); however, the pharmacokinetics of AGuIX has not been fully understood. To quantitatively determine the dose effect for radiation therapy, it is critical to define the biodistribution and pharmacokinetics of these nanoparticles. ^64^Cu, one of the most commonly used radioisotopes in positron emission tomography (PET), has decay characteristics (*T*_1/2_ = 12.4 h) that provide it with the flexibility to image small molecules and large slow-clearing proteins and nanoparticles. In this study, we radiolabeled AGuIX with ^64^Cu for the initial evaluation of its in vivo biodistribution in HepG2 tumor-bearing nude mice to more accurately measure the magnitude and duration of its retention within the tumor microenvironment. To further conduct proof-of-concept studies with AGuIX as a radiation sensitizer in HepG2 tumor-bearing nude mice, we used ^18^F-FDG PET/CT, a clinically proven imaging technology for tumor metabolism to monitor therapy response and to evaluate the glucose metabolism of the HepG2 tumor before and after radiotherapy with or without AGuIX.

## Methods

### General Information

Dehydrated, spherical, and sub-5 nm gadolinium nanoparticles (AGuIX) were obtained from Nano-H (Lyon, France) and used without purification. The nanoparticles consist of gadolinium atoms attached to a polysiloxane shell via built-in DOTA chelators. Nanoparticles were rehydrated in sterile, DEPC-treated water (Invitrogen, USA) and stored at 4 °C until use according to manufacturer’s instructions. Megestrol acetate was purchased from Sigma Chemical Co., (St. Louis, MO, USA). The human HCC cell line, HepG2, was obtained from American Type Culture Collection (American Type Culture Collection, University of Virginia, VA, USA). ^64^Cu isotope was purchased from Wisconsin University. Other chemicals and reagents were purchased from Sigma Chemical Co., (St. Louis, MO, USA) and used without further purification or processing. Six-week-old male BALB/c athymic nude mice weighing between 16 and 18 g were purchased from Charles River. The animal study was approved by the Institutional Animal Care and Use Committee at the University of Virginia.

### Transmission Electron Microscopy (TEM)

AGuIX nanoparticles at a concentration of 0.5 mM in the above solutions were incubated with HepG2 cells for 1 h [10]. Then, the residual nanoparticles were washed with 0.1 M of phosphate buffer saline and purified by centrifugation. Cell pellets with nanoparticles were stained with 4% formaldehyde and 1% glutaraldehyde in 0.1 M Pb for imaging.

### ^64^Cu Radiolabeling

AGuIX nanoparticles were radiolabeled with the ^64^Cu isotope. We first mixed 200 μl of AGuIX nanoparticle solution (10 μmole of AGuIX) with 100 μl of 0.5 M of NH_4_OAc buffer (pH = 5.5). After incubation for 5 min, 1–3 mCi of ^64^CuCl_2_ in 0.1 N of HCl were added, and the reaction mixture was incubated at 37 °C for 1 h. The reaction mixture was then sterilized by filtration through a 3k Amicon Ultra Centrifugal Filter (Merck Millipore). The radiochemical purity was determined by iTLC using 20 mM of citric acid as the mobile phase as previously described [11].

### Tumor Models

HepG2 cells were grown in MEM containing 1 mM of sodium pyruvate, 1 mM of nonessential amino acids, and 10% FCS (Life Technologies, Inc., Grand Island, NY, USA). Cells were maintained in a humidified atmosphere of air/CO_2_ (19/1), and they were sub-cultured every 2–3 days.

HepG2 cells (5 × 10^6^) were collected in 0.1 ml of HBSS, and these cell suspensions were then injected subcutaneously into the right flank of each nude mouse using a 27-gauge needle. The ears of the nude mice that received cell injections were tagged for identification. Generally, solid tumors started to become visible 2 weeks after the injection of HepG2 cells.

### Biodistribution in Tumor-Bearing Mice of ^64^Cu-AGuIX

Tumor-bearing nude mice (5 male and 4 female mice) were randomly divided into three groups and injected intraperitoneally with the ^64^Cu-AGuIX, with an activity of approximately 0.9 MBq, in a volume of 0.2 mL. The mice were sacrificed by cervical dislocation under anesthesia with isofluorane inhalation at 9, 21, and 40 h after injection. The organs of interest (the heart, muscle, lung, kidney, spleen, liver, and tumor, etc.) were dissected and weighed, and 100 μL of blood was taken from the ventricular cavity. The activity for each sample was determined by using a γ counter (CRC-7, Capintec Inc., NJ, USA). The distribution of the radioactivity in different tissues and organs was calculated and expressed as the percentage of injection dose per gram (% ID/g).

### Micro-PET Imaging of ^64^Cu-AGuIX in Nude Mice

The ^64^Cu-AGuIX (22.2 MBq) in 0.2 mL solution of saline was injected intraperitoneally in each tumor-bearing nude mice. Each animal was placed prone on the bed of a PET system (SuperArgus, Sedecal, Spain). The PET images were acquired for different time periods at 9 and 21 h post-injection of ^64^Cu-AGuIX under the anesthesia of 4–5% isoflurane for induction and 1–2% for maintenance, both balanced by oxygen.

### Irradiation Setup and ^18^F-FDG PET Evaluation of Xenografts

For PET imaging studies to evaluate the radiosensitization of AGuIX during radiation therapy, 12 nude mice bearing HepG2 tumors were divided into three groups, with four mice randomly assigned per group. For the baseline PET imaging, the mice were injected with ^18^F-FDG (16.4 ± 4.7 MBq) through the tail vein and kept under general anesthesia for 10 min of PET static imaging at 30 min p.i. (post-injection) with a small-animal PET scanner (Madiclab, Shandong, CN). PET images were reconstructed using the 3D OSEM algorithm, a voxel size of 0.91 × 0.90 × 0.90 mm, and a spatial resolution at the center of the field of view of 1.3 mm.

For the irradiation study, each group received injections by the tail vein with 0.1 mL of normal saline, 1 mg (0.1 mL) of AGuIX, and 10 mg (0.1 mL) of AGuIX. At 1 h after injection, these nude mice were irradiated using an X-ray source (X-RAD 320, Precision X-Ray, North Branford, CT, USA), which was operated at 250 kV and 8 mA, with a 2-mm Al filter at a dose rate of 1.2 Gy/min for a total dose of 6 Gy. On the following day, the same irradiation protocol was repeated with the mice. At 1 day after two irradiation treatments, these mice were imaged with ^18^F-FDG (11.1 ± 1.0 MBq) PET using the same protocol as the first PET scan. Standard uptake value max (SUVmax) was determined by drawing regions of interest (ROIs) in the tumor areas (Madiclab, Shandong, CN).

### Statistical Analysis

All of the experiments were carried out in triplicate and the results expressed as the mean ± standard error (SE). Statistically significant differences were calculated using a two-tailed unpaired *t* test or one-way analysis of variance; *p* values of < 0.05(*) and < 0.01(**) were considered to be significant.

## Results and Discussion

While contrast-enhanced MRI has been extensively used in AGuIX-based image-guided radiotherapy, the detection limit of the nanoparticles’ concentration is a concern due to the longer time measurement of the pharmacokinetics of the nanoparticles. With a much more sensitive and higher quantitative capacity, PET extends the dynamic range of concentrations to much lower nanomolar concentrations that are undetectable by contrast-enhanced MRI. In this report, we described the labeling and evaluation of the biodistribution and pharmacokinetics of AGuIX with ^64^Cu for potential PET imaging-guided radiotherapy.

### TEM Study

**Fig. 1:**
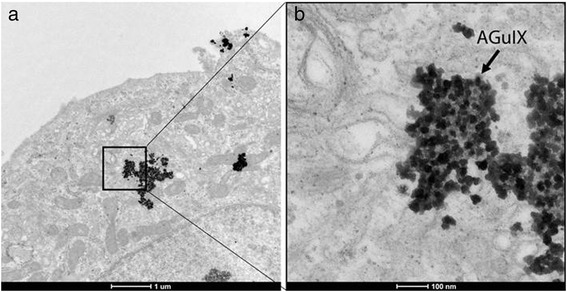
Localization of AGuIX within HepG2 cells. **a**. TEM images (× 6500) depict the uptake of AGuIX into the HepG2 cells. **b**. Magnified TEM image (× 52000) shows the distribution of AGuIX nanoparticles in the cytoplasm

For cell incubation studies, a concentration of 0.5 mM of AGuIX nanoparticles was chosen based on published data and AGuIX nanoparticles were incubated with HepG2 cells for 1 h [10]. Uptake into the cytoplasm of HepG2 cells was observed (Fig. 1). This result is in agreement with previously published studies in which AGuIX nanoparticles were incubated with other types of cell lines [12, 13]. We also observed that AGuIX showed an excellent dispersion shape in the HepG2 cells, suggesting that AGuIX was stable in the cells.

### Radiolabeling

The labeling was conveniently carried out with the current form of AGuIX with the built-in chelator DOTA in one step for > 98% radiochemical yield. Using the iTLC test to identify the radiolabeled nanoparticles that were retained in the original spots, the labeling resulted in specific activity and radiochemical purity of approximately 3–10 MBq/μmol and 98%, respectively. An average 50–100 MBq of the final product was obtained by each synthesis.

### Biodistribution Studies

**Fig. 2:**
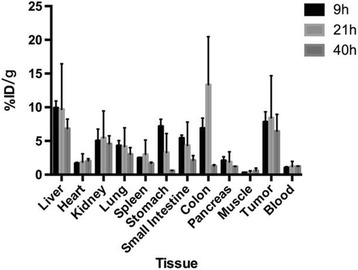
Biodistribution of ^64^Cu-AGuIX in nude mice bearing HepG2 tumors. Radioactivity uptake in each tissue/organ were presented in %ID/g at 9, 21, and 40 h after intraperitoneal injection of ^64^Cu-AGuIX (mean ± SD, *n* = 3)

^64^Cu-AGuIX nanoparticles were injected intraperitoneally, the biodistribution was determined in HepG2 tumor-bearing nude mice, and it was compared with previously reported ones. As shown in Fig. 2, the biodistribution in each organ/tissue is presented as the percentage of administered activity (injected dose) per gram of tissue (% ID/g). The results clearly showed that ^64^Cu-AGuIX accumulated in the tumor with excellent retention from 9, 21, and 40 h p.i. with the uptake of 7.82 ± 1.50, 8.43 ± 6.23, and 6.84 ± 1.40% ID/g, respectively. This long-term retention may be attributed to the uptake of the AGuIX nanoparticles inside the cells and thus is related to ^64^Cu residence in the cells. In agreement with other reports [11, 14], although different isotope radiolabeling and injection routes were used, the ^64^Cu radiolabeled nanoprobes exhibited a much lower uptake (lower than 1% ID/g) in other normal organs and tissues and fast clearance. Collectively, these data suggest the potential use of ^64^Cu-labeled AGuIX as a tool to measure the biodistribution and pharmacokinetics of AGuIX to help guide the plan of radiotherapy in which these nanoparticles are used as radiosensitizers. In this study, the kidney uptake is much lower than others have reported because this study used intraperitoneal injection [11, 14].

### Micro-PET Imaging in Nude Mice

**Fig. 3:**
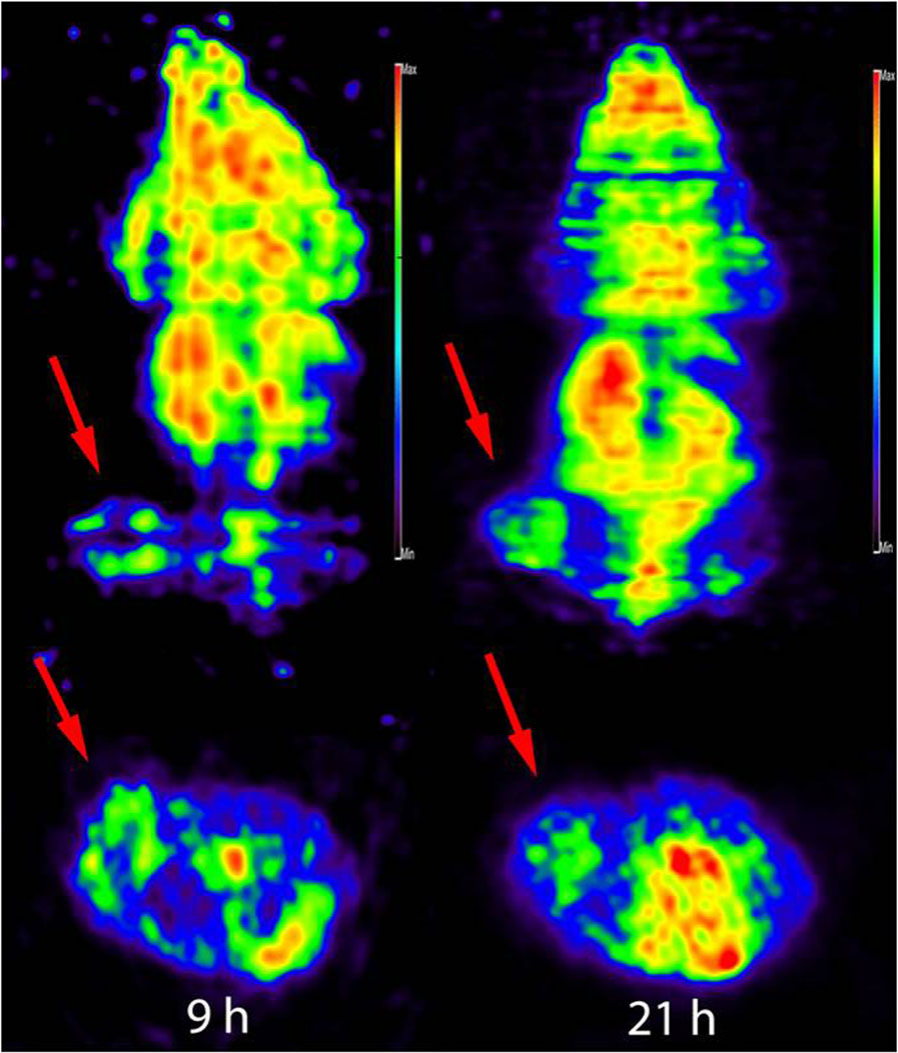
Micro-PET images of tumor mice. PET images (upper, coronal view; bottom, transverse view) of nude mice with tumors (*red arrow*) were acquired at 9 h (left) and 21 h (right) after intraperitoneal injection of ^64^Cu-AGuIX

Micro-PET imaging showed that high uptake of ^64^Cu-AGuIX was observed in tumor, kidneys and liver in tumor-bearing nude mice (Fig. 3). The tumor was clearly visible after administration of ^64^Cu-AGuIX at 9 h and even be more clearly until 21 h post-injection as the background decrease.

### ^18^F-FDG PET/CT Evaluation of Irradiated Xenografts with or Without AGuIX

**Fig. 4:**
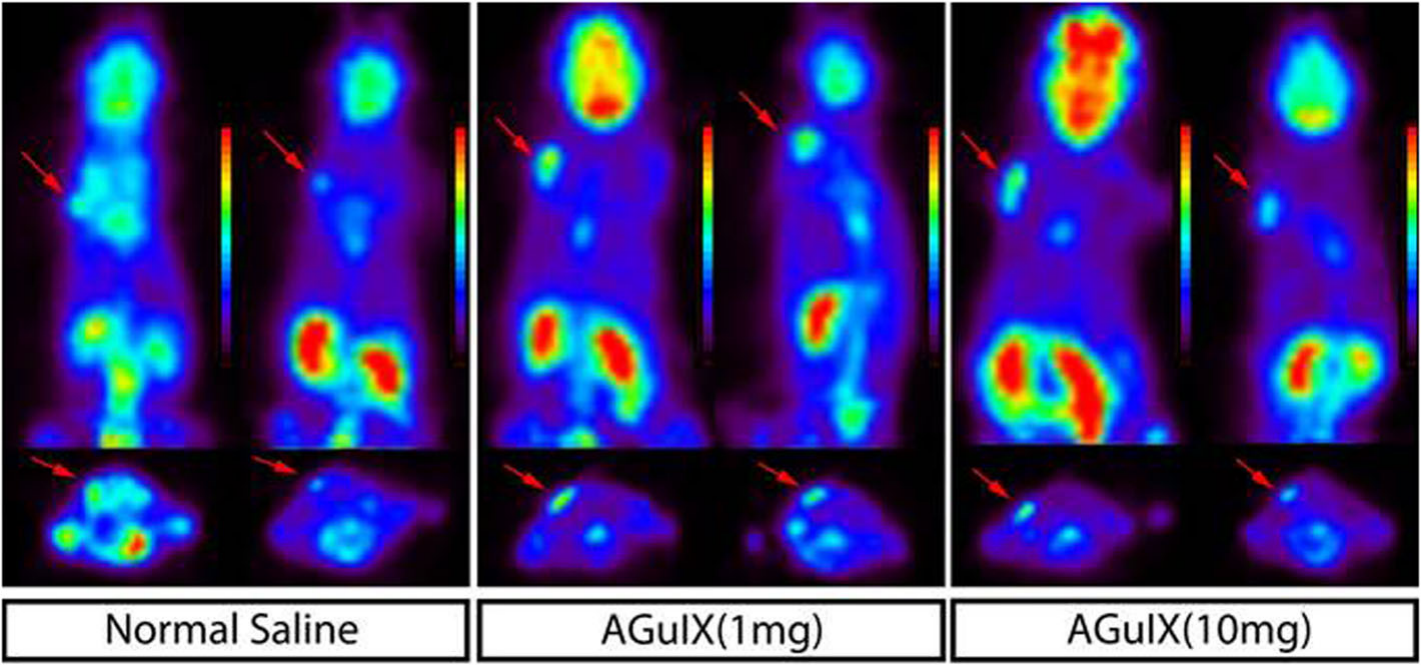
^18^F-FDG PET images of the mice before and after radiation. ^18^F-FDG PET images were compared in each panel before (left) and 1 day (right) after irradiation, and the three panels showed the images of mice injected by tail vein injection of normal saline (left panel), 1 mg of AGuIX (middle panel), and 10 mg of AGuIX (right panel), respectively. The same color scale was applied to each of the images

**Fig. 5:**
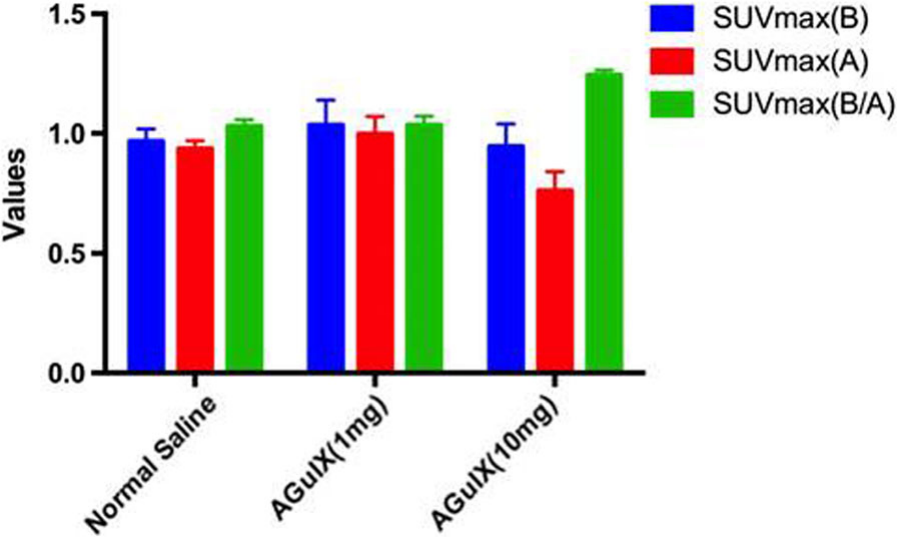
^18^F-FDG PET quantitative evaluation before and after irradiation. T/L (B), the ratio of SUVmax (tumor) to SUVave (liver) before irradiation; T/L (A), the ratio of SUVmax (tumor) to SUVave (liver) after irradiation; T/L (B/A), the ratio of T/L (B) to T/L (A); AGuIX (1 mg), 1 mg of AGuIX injected; AGuIX (10 mg), 10 mg AGuIX injected

To evaluate the different radiotherapy responses with or without AGuIX administration, ^18^F-FDG PET/CT imaging was performed to monitor the metabolic changes after irradiation with or without AGuIX injection at two different dosages. The decrease in ^18^F-FDG uptake in the xenografts was observed in all of the irradiated mice (Fig. 3). SUVmax (B/A), the primary indicator of effectiveness of radiosensitization, was 1.03 ± 0.03, 1.04 ± 0.04, and 1.24 ± 0.02 for the mice receiving normal saline, 1 mg of AGuIX, and 10 mg of AGuIX, respectively (Fig. 4). For the 10 mg of AGuIX group, T/L (B/A) was significantly increased compared with the 1 mg of AGuIX group (*p* < 0.001, independent sample test) and with the normal saline group (*p* < 0.001, independent sample test). There was no significant difference for T/L (B/A) between the groups receiving 1 mg of AGuIX and normal saline (*p* = 0.83, independent sample test) (Fig. 5). These results suggest that the glucose metabolism of the xenografts was suppressed mostly in the irradiated mice that received a 10-mg injection of AGuIX; although the radiotherapy may induce inflammation which can also lead to FDG uptake. In this study, we choose the same radiation dose and the same time point post-RT therapy for all the groups to offset any systemic errors. Therefore, the extent of the inflammation caused by RT should be about the same for all three groups and the contribution to FDG uptake induced by the inflammation should be about the same level too. Other PET imaging probes may be used to avoid this concern. Nonetheless, these findings provide proof-of-concept that AGuIX can be used as a tumor radiation sensitizer in HepG2 tumor-bearing mice.

Last, the radiation dose absorbed from the nuclear imaging probe itself is also a critical concern when considering a move into clinical use. The current form of AGuIX nanoparticles has been thoroughly investigated for metabolism and toxicity in vivo and approved for human studies by the FDA [15]. By labeling either ^68^Ga (~ 1 h decay half-life) or ^89^Zr (78 h decay half-life), the results of the biodistribution study in mice by intravenous injection showed an extremely high uptake in the kidneys at over 20% ID/g from as early as 30 min by ^68^Ga to as late as up to 72 h by ^89^Zr [11, 14]. Although fast excretion by the kidneys is generally beneficial, due to the sensitivity of the kidneys to radiation, whether the kidneys can tolerate this high uptake and the mechanism of retention for such a long period of time are unknown. In this study, the kidney uptake was at ~ 5% ID/g, lower than that in the liver and tumor for the whole period of the investigation. This difference might be assumed to be due to the different injection routes. By intraperitoneal injection, the AGuIX nanoparticles were continuously absorbed by the peritoneum, whereas by intravenous injection, the nanoparticles were excreted quickly by the kidneys. Because the radiation sensitivity of each organ and tissue is different, the final determination of the radioactive theranostic probes for translation into clinical use needs further detailed dosimetry studies.

## Conclusions

AGuIX nanoparticles have been successfully labeled with ^64^Cu with a high yield. The biodistribution studies indicated that the radiotracer ^64^Cu-AGuIX displayed high accumulation in tumors and was retained for a long period in the HepG2 xenograft of the nude mice, suggesting that they are potential theranostic nanoprobes for image-guided radiotherapy in HCC. The significant reduction of ^18^F-FDG uptake after radiotherapy in the group of tumorous nude mice injected with AGuIX provided evidence that AGuIX can be used as a tumor radiation sensitizer to enhance radiotherapy in HepG2 tumor-bearing mice. Further investigations on the dosimetry are needed to determine the radiation toxicity for potential translation into clinical applications.
